# Autoimmune Demyelinating Polyneuropathy as a Manifestation of Chronic Graft-versus-Host Disease after Adult Cord Blood Transplantation in a Patient with Chronic Lymphocytic Leukemia

**DOI:** 10.1155/2014/758094

**Published:** 2014-09-14

**Authors:** Fredrick Hogan, Melhem Solh

**Affiliations:** ^1^Blood and Marrow Transplant Center, Florida Hospital Cancer Institute, 2501 N Orange Avenue, Suite 581, Orlando, FL 32804, USA; ^2^University of Central Florida, 6850 Lake Nona Boulevard, Orlando, FL 32827, USA

## Abstract

Immune mediated demyelinating disease after allogeneic stem cell transplantation is a rare entity with unclear etiology. Acute inflammatory demyelinating polyneuropathy (AIDP) has been reported after related and adult unrelated allogeneic stem cell transplantation but no such case has been reported after unrelated cord blood transplantation. We hereby present the first case of AIDP after double umbilical cord blood transplantation (DUCBT). A 55-year-old man with chronic lymphocytic leukemia (CLL) received a cord blood transplant for relapsed refractory disease with high risk cytogenetics. On day 221, patient presented with skin rash, tingling in both lower extremites, and ascending paralysis that progressed rapidly over the course of 2 days. The workup resulted in a diagnosis of AIDP and administration of intravenous immunoglobulins plus steroids was initiated. Motor and sensory powers were fully recovered and his chronic GVHD was managed for several months with single agent sirolimus.

## 1. Introduction 

Cord blood transplantation is an acceptable treatment modality for adult patients with high risk malignancy lacking a suitable matched sibling or adult unrelated donor. Autoimmune diseases occurring after allogeneic hematopoietic cell transplantation (HCT) are mostly antibody mediated and organ specific [1, 2]. Neurologic complications after allogeneic HCT occur in 14–42% of patients [3, 4] and can include seizures, encephalopathy, infections, and polyneuropathy. Immune mediated demyelinating disease after HCT is a rare entity with unclear etiology that can be a manifestation of graft-versus-host disease [5]. A thorough workup is always warranted to rule out infectious etiologies when patients present with neurologic manifestations after allogeneic HCT and in particular cord blood transplantation.

## 2. Case Presentation

A 55-year-old male with relapsed refractory CLL received double cord blood transplant (DUCBT) with two 5/6 HLA matched cord blood units (antigen levels HLA-A, HLA-B and allele level HLA-DRB1). Conditioning was a reduced intensity regimen consisting of fludarabine, Cytoxan, and total body irradiation. The treatment for prevention of graft-versus-host disease (GVHD) was with cyclosporine and mycophenolate. On day 13 after DUCBT, he developed upper and lower respiratory tract infection with respiratory syncytial virus (RSV) requiring inhaled ribavirin therapy. Patient achieved a successful neutrophil engraftment by day 27 after DUCBT. Early posttransplant course was complicated by grade 4 acute GVHD of the gut with a complete resolution with steroid therapy and successful taper of all immunosuppression by day 180 after DUCBT. On day 221 after transplantation, patient presented with skin rash and tingling in both feet that progressed rapidly to lower extremity paralysis over the course of 2 days. Physical exam showed maculopapular rash affecting his upper extremities, upper chest, and back area accounting for almost 50% of his body surface area. Neurologic exam was significant for symmetric motor weakness in lower extremities 2/5, plantar flexion, and knee flexion 3/5. He had loss of deep tendon reflexes in both lower extremities (Achilles and Patellar) and upper extremities (biceps and triceps). Laboratory workup revealed normal blood counts, organ function (kidney and liver), vitamin B12, folate, thyroid function (TSH level), and free cortisol. Serum electrophoresis and immunofixation were also normal. Magnetic resonance imaging of the central nervous system showed mild neural foramina narrowing at the L4-L5 level. Serologies for Lyme disease, Epstein Bar virus (EBV), syphilis, Cytomegalovirus (CMV), Hepatitis profile, HIV, toxoplasma, enterovirus, and human herpes virus 6 were all negative. Blood tests for autoimmune markers including (anti-nuclear antibody) ANA, acetylcholine esterase, and volted calcium channel antibodies were normal. A lumbar puncture was performed and showed a high protein level of 67 mg/dL, 1 nucleated cell/mm^3^, and a normal glucose level. Cerebrospinal fluid was negative for oligoclonal bands, West Nile virus, cryptosporidium, HHV6, herpes viruses 1 and 2, gram stain, and cultures. Nerve conduction studies and needle electromyography were suggestive of acute demyelinating polyneuropathy. A skin biopsy was consistent with GVHD.

Based on the above workup, he was diagnosed with AIDP and started on therapy with intravenous immunoglobulins at 0.5 gm/kg for 4 days and prednisone 1 mg/kg daily for the treatment of GVHD. Etiology of AIDP was presumed to be related to GVHD as his workup was negative for campylobacter, HIV and CMV, and other infectious etiologies. He improved significantly over the next 4 weeks and became ambulatory without assistance but his weakness symptoms relapsed as his prednisone dose was being tapered. Prednisone was increased again to 1 mg/kg and sirolimus was started. Patient was successfully tapered off prednisone and remained fully ambulatory without assistance or evidence of GVHD on single agent sirolimus. Sirolimus was eventually discontinued at 18 months after DUCBT with no further relapse in the neurologic manifestations.

## 3. Discussion

Neurologic and immune complications after allogeneic HCT are relatively common [1–5] but immune demyelinating diseases are rare and are seen in only 0.5% of allogeneic recipients [4]. The extent of autoimmune diseases after cord blood transplantation was recently reported through a retrospective analysis from the Eurocord registry [6]. In this Eurocord analysis, fifty-two out of 726 cord blood recipients developed at least 1 autoimmune disease with a cumulative incidence of 5% at one year after transplantation. Most of the autoimmune events were directed against the hematopoietic system with few cases affecting other organs (thyroiditis, psoriasis, colitis, arthritis, and glomerulonephritis). Six of the 52 patients who developed autoimmune complications ended up dying as a result of these complications. Of note, there were no autoimmune neurologic manifestations in this large retrospective series [6].

Multiple case reports have described immune mediated demyelinating disease in the central nervous system after HCT and included optic neuritis, myelitis, or a combination of both [5, 7, 8]. The involvement of the peripheral nervous system was also described after allogeneic and autologus HCT [9, 10]. A retrospective analysis from Memorial Sloan-Kettering cancer center evaluated the incidence and risk of immune mediated demyelinating diseases (IMDD) after allogeneic HCT [4]. Among 1484 HCT recipients, the incidence of IMDD was 0.5% occurring at a median of 4 months and never later than 13 months after HCT. Three of the affected patients had AIDP and all were successfully treated with intravenous immunoglobulins with or without rituximab.

The pathogenesis of AIDP is not well known. It is believed that GVHD may be a possible mechanism but many patients who develop AIDP do not have manifestations of other organs being involved with GVHD [4]. Our patient had manifestation of GVHD in his skin at the time of neurologic presentation and had an excellent response to immunosuppression throughout his treatment course. Unfortunately, no nerve or muscle biopsy was obtained during the initial presentation to assess for CD8+ T cell infiltration similar to what is seen in tissues affected by GVHD.

In conclusion, AIDP is a rare complication after allogeneic HCT and can present secondary to GHVD. This case documents that AIDP as a manifestation of GVHD can occur after adult cord blood transplantation and can be successfully treated with intravenous immunoglobulins and immunosuppression.
